# Interspecies and Intrastrain Interplay among *Leishmania* spp. Parasites

**DOI:** 10.3390/microorganisms10101883

**Published:** 2022-09-21

**Authors:** Bruna Dias das Chagas, Thaís Martins Pereira, Lilian Motta Cantanhêde, Gabriela Pereira da Silva, Mariana Côrtes Boité, Luiza de Oliveira Ramos Pereira, Elisa Cupolillo

**Affiliations:** Leishmaniasis Research Laboratory, Oswaldo Cruz Institute, FIOCRUZ, Rio de Janeiro 21040360, Brazil

**Keywords:** *Leishmania*, coinfections, mixed infections, coculture, hybrid, intercellular communication

## Abstract

*Leishmania* parasites present astonishing adaptative abilities that represent a matter of life or death within disparate environments during the heteroxenous parasite life cycle. From an evolutionary perspective, organisms develop methods of overcoming such challenges. Strategies that extend beyond the genetic diversity have been discussed and include variability between parasite cells during the infections of their hosts. The occurrence of *Leishmania* subpopulation fluctuations with variable structural genomic contents demonstrates that a single strain might shelter the variability required to overcome inconsistent environments. Such intrastrain variability provides parasites with an extraordinary ability to adapt and thus survive and propagate. However, different perspectives on this evolution have been proposed. Strains or species living in the same environment can cooperate but also compete. These interactions might increase the replication rate of some parasites but cause the loss of more aggressive competitors for others. Adaptive responses to intra- and interspecific competition can evolve as a fixed strategy (replication is adapted to the average genetic complexity of infections) or an optional strategy (replication varies according to the genetic complexity of the current infection). This review highlights the complexity of interspecies and intrastrain interactions among *Leishmania* parasites as well as the different factors that influence this interplay.

## 1. Introduction

Few reports have described infections by more than one *Leishmania* species, which is likely because of the lack of efficient diagnostic methods for cases of this nature. Therefore, the clinical and epidemiological impact of mixed infections remains to be explored, and the technical limitations for such studies must be overcome.

Direct parasitological examination by visualization of amastigotes in clinical specimens, which are used routinely in the diagnosis of leishmaniasis, does not allow for the identification of mixed infections because the number of morphological differences is insufficient to differentiate species. In turn, assays carried out after parasite isolation and cultivation do not always allow for the identification of mixed infections because in vitro maintenance may favor a particular species or even a specific strain. The introduction of molecular methods for the diagnosis of leishmaniasis and *Leishmania* typing applied directly to clinical specimens enables mixed infection detection. Nevertheless, the results will be affected by the sensitivity of the methodology employed and the parasite burden for each species.

In nature, mixed infections by *Leishmania* species are naturally present in vectors, reservoirs, and humans. Experimental coinfections have contributed to a better understanding of the mechanisms involved in these interactions and their consequences. For instance, mixed infections by different species of *Leishmania* can alter the transmission dynamics, as observed in gerbils infected by *L. major* and *L. turanica* [1]. Experimental mixed infections with these two species in *Phlebotomus papatasi* sand flies showed that although they coexist in the vector, the prevalence of each species may vary as a consequence of the shared environment [2]. In a human monocyte cell line previously infected with a *Leishmania* species, infection by another species is not impaired, even if the second species was added 3 h later [3]. A recent study of experimental coinfections using a hamster model (*Mesocricetus auratus*) infected with *L. (L.) amazonensis* and *L. (L.) infantum* performed clinical, histopathological, and immunological analyses and showed that mixed infections are associated with more severe clinical manifestations than single infections [4]. A case of human mucosal leishmaniasis (ML) involving coinfection by *L. (L.) tropica* and *L. (L.) major* was also reported [5], and mixed infections by different strains of *L. infantum* have been shown to influence the therapeutic response [6].

Inter- and intraspecies interactions occur among *Leishmania* parasites in both vertebrate and invertebrate hosts; genetic exchange has already been demonstrated for these parasites, and it mainly occurs during the development stage in sandflies. Putative hybrids and genetic recombination have been reported in different studies focused on *Leishmania* typing and genetic analyses of natural populations. The impact of this reproductive strategy is a current focus of debate [7]. Mosaic aneuploidy is an important feature in discussions of multiple infections in *Leishmania*, and in *Leishmania* parasites, this feature implies that each strain is already in a mixture, which represents a genetic adaptation strategy of this parasite [8]. Mosaic aneuploidy refers to a variation in the number of homologous chromosomes per cell within a subpopulation of cells. For example, 30% of cells presenting chromosome 2 in a diploid state, 20% as triploid and 50% as tetraploid

Thus, studies of mixed infections can ultimately contribute to elucidating the complex molecular basis of clinical and epidemiological features and the parasite-specific factors that might lead to the worsening or improvement of infections. The molecular mechanisms involved in the *Leishmania*–*Leishmania* interaction process have not been widely explored; however, they are essential for maintaining a microbial community [9] either by direct contact, media sharing or both. Moreover, the diversity of these communities has a profound impact on the biology of the parasite and, consequently, on the interaction of *Leishmania* with its vertebrate and invertebrate hosts. In vitro promastigote cultures have been investigated, and the in vitro growth of *L. mexicana* has been shown to be impaired when grown together with *L. amazonensis* [10]. The cocultivation of *L. amazonensis* strains with a distinct susceptibility profile to pentamidine demonstrated that in vitro growth depends on the interaction between strains that share the same environment [11]. The growth of *L. donovani* cocultured with *Trypanosoma brucei* was severely affected, and swelling and lysis were observed. In these cases, direct contact was necessary because *T. brucei* did not hinder growth when they were physically separated [12]. A species or strain may produce more than one agonist or antagonist element with different physicochemical and biological properties, which will depend on environmental conditions among other factors.

Studies with bacteria and fungi performed to characterize the biofilm secretome synthetized by a single or distinct species showed an increase in proteins secreted by mixed biofilms, thus reflecting the competition for iron by the microorganisms [13]. *Leishmania* promastigotes secrete proteins involved in immunomodulation, signal transduction and intracellular survival, such as HSP70, acid phosphatase, protein kinase C receptor (LACK), elongation factor 1, and triparedoxin peroxidase [14]. In addition, in vitro-secreted vesicles, exosomes and ectosomes carry various molecules, including GP63 surface metalloproteases, which is a critical parasite virulence factor. The characterization of exosomes produced by in vitro promastigotes indicates that they are similar to those observed during interactions with the vector insect [15]. Studies with trypanosomatids have shown the role of these vesicles in the process of parasite interactions, although their role in *Leishmania*–*Leishmania* interactions has not yet been investigated [16].

Some mechanisms allow a population of individuals to coordinate global behavior and act as a multicellular unit, which is a phenomenon known as “quorum sensing”, a process of cell–cell communication that allows microorganisms such as bacteria to share information, such as cell density, and respond by adjusting gene expression, consequently changing phenotypes. Quorum sensing is a poorly explored mechanism in *Leishmania*, which is a parasite with high genetic and phenotypic variability and with tremendous adaptive ability. An evaluation of *T. congolense* demonstrated that this parasite has a growth control mechanism based on density and that the interaction with other species of trypanosomes is dependent on quorum sensing [17].

**Figure 1:**
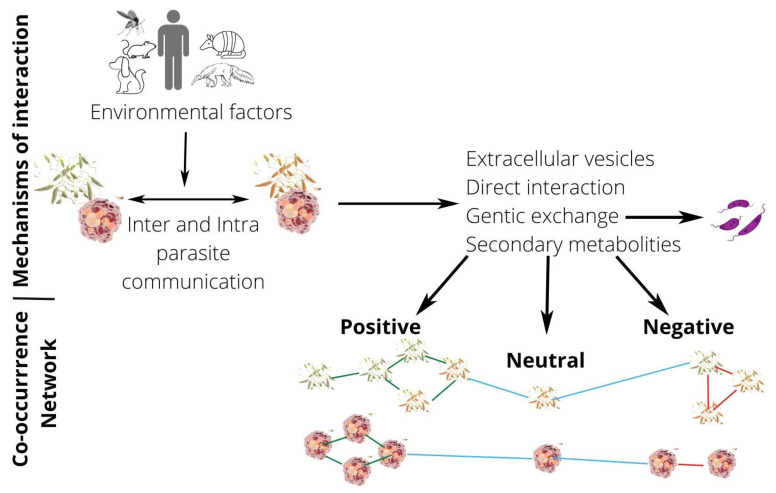
**Picture of *Leishmania* interactions through co-occurrence networks.** *Leishmania* interactions are influenced by hosts’ factors (environmental factors) and can result in positive, neutral, and negative interactions types. In co-occurrence networks, nodes are represented by promastigotes (in invertebrate hosts) or amastigotes (in vertebrate hosts) of *Leishmania* spp., and edges are representing associations between parasites (nodes). Blue edges indicate neutral interactions, green edges indicate positive interactions, while red edges suggest negative interactions between *Leishmania* species, strains or genotypes.

This review article presents published data on natural and experimental mixed infections by distinct *Leishmania* species or strains and addresses the complexity of interspecies and intrastrain interactions in *Leishmania* parasites and the various factors influencing this relationship (Figure 1).

Table 1 presents a summary of studies reporting inter- and intraspecies interactions among *Leishmania* parasites in their hosts or in experimental conditions in vitro and in vivo.

## 2. Natural Mixed Infections by *Leishmania* Species

Leishmaniasis is a complex disease that is endemic in large areas of the tropics, subtropics and Mediterranean basin, globally spanning more than 98 countries. The disease is caused by several *Leishmania* species, an obligate protistan parasite, and the transmission cycles involve different vertebrate and sandfly species. According to the World Health Organization [47], there are three main forms of this disease: visceral (also known as kala-azar), cutaneous (the most common), and mucocutaneous.

Despite some taxonomic controversies, there are more than 30 recognized *Leishmania* species found in mammals and reptiles, and one is found in sandflies only. At least 20 are pathogenic to humans across the world (Table 2). While some *Leishmania* species are geographically restricted to an endemic area, others are widespread. For example, in the Amazon region [48], a greater number of species coexist in sympatry, and some are only found there [49]. Importantly, although not yet well defined, different clones or strains may also be observed in this transmission environment. Such an intricate epidemiological scenario can contribute to the complexity of the disease assuming the possibility of mixed infections caused by different subpopulations, species or strains of *Leishmania*.

Mixed infections by *Leishmania* species are very likely to be underdiagnosed; nevertheless, coinfected human patients have been reported in the literature. Infection outcome is influenced by the *Leishmania* species involved, and multiple strains, genotypes or species infections are expected to impact host–parasite relationships. In mammalian hosts, the *Leishmania* infection profile is characterized by cytokine and chemokine production and may be related to the species of *Leishmania* as well as other factors [89]. Despite the host’s immunological competence, data from the literature indicate an essential role of the *Leishmania* species over the course of infection. In the *Leishmania* (*Viannia*) subgenera, for example, two species that cause tegumentary leishmaniasis may lead to different manifestations of the disease: *L. naiffi*, which is commonly associated with low virulence in cutaneous lesions [90,91] and the *L*. *braziliensis*, which is correlated with mucosal and atypical lesions frequently refractory to treatment [92,93,94,95]. In the *Leishmania* (*Leishmania*) subgenera, this polarization is also observed in infections caused by *L*. *tropica* and *L*. *major*, which lead to small or no lesions and severe lesions, respectively [96,97,98]. Determining how coinfecting *Leishmania* species interact with their hosts is not trivial. Within-species interactions can be direct (e.g., via resource competition) or indirect (e.g., via immunomodulation). Experimental infections in rhesus macaques indicate cross-reacting immune responses and possible cross-protection between taxonomically different *Leishmania* parasites [24]. Although existing data cannot be used to predict coinfection scenarios with different *Leishmania* species, such scenarios should be considered due to the potential complications in the course of the disease and response to the treatment.

A few reports have associated coinfection with therapeutic response or atypical clinical manifestations, although the differences relative to infection with the separate agents have not been presented. For example, a survey of Bolivian patients showed that 27.6% presented mixed infections by different species of *Leishmania* and 13.8% presented mixed infections by *Leishmania* species and *T*. *cruzi* [23], which significantly influenced the therapeutic outcomes. Treatment failure was linked to mixed infection by two *L. infantum* zymodemes, which present distinct biological behavior and different sensitivities to meglumine antimoniate [6]. Mixed infection by *L*. *amazonensis* and *L*. *infantum* was associated with diffuse cutaneous leishmaniasis (DCL) in a Bolivian patient. The lesion presents atypical characteristics, possibly due to coinfection, and abundant parasites and vacuolated histiocytes were observed, which is compatible with DCL diagnoses [22]. Atypical disseminated leishmaniasis was associated with mixed infection caused by *L*. *guyanensis* and *L*. *amazonensis*. Over the course of the infection, a mixed patterns of clinical, histopathological and immunological characteristics related to the two species were observed, such as the absence of cellular response and failure of therapy, which are consistent with *L*. *amazonensis* infection, and multiple concomitant lesions with a low antibody titer, which are consistent with *L*. *guyanensis* infection [40]. In Iran, a patient presenting mucosal leishmaniasis was reported to have nasal and oral lesions caused by *L*. *major* and *L*. *tropica*, respectively. The nasal lesions appeared before the oral lesions, suggesting that previous infection by *L. major* did not protect against *L. tropica* in this patient [5]. Conversely, a study using BALB/c mice reported that primary infection with *L. tropica* induces partial protection against *L. major* infection [26]. However, the further clinical progression of a subclinical infection associated with *L. tropica* in the Iranian patient after *L. major* infection cannot be excluded. Similarly, protection against *L. braziliensis* was not observed for a patient presenting coinfection with *L. infantum* (named *L. donovani* in that work) [19], and cutaneous lesions appeared after visceral leishmaniasis clinical signs; however, subclinical infection cannot be disregarded.

It is worth mentioning the mixed infections reports that do not address complications due to mixed infections. Typical cutaneous lesions caused by the coinfection of *L. braziliensis* and *L. amazonensis* were observed in a patient from the Brazilian Amazon region [18], and cutaneous leishmaniasis caused by a mixed natural infection by *L*. *braziliensis* and *L*. *lainsoni* has been demonstrated in a Peruvian patient, who showed a good response to treatment with sodium stibogluconate and no evidence of mucosal involvement [33].

The simultaneous presence of *L*. *donovani* and *L*. *major* in typical localized cutaneous ulcers of leishmaniasis patients from Sudan was reported, and no evidence of visceralization was observed [34]. The same coinfection was reported in a case from Iraq, although in this case, the patient presented concomitant visceral and cutaneous leishmaniasis [21]. Patients clinically diagnosed with visceral leishmaniasis presented mixed infection by *L. donovani* and *L. major* in the spleen, and the mixed cultures obtained from the tissue fragments were inoculated in laboratory animals, producing both visceral and cutaneous leishmaniasis [20].

Atypical clinical manifestations were observed in immunocompromised patients presenting infection by two *Leishmania* species. An atypical cutaneous lesion caused by *L*. *donovani* was observed in a Brazilian HIV-positive patient after long-term evidence was obtained of the visceral parasite *L*. *infantum* in the bone marrow [30]. A case of disseminated cutaneous leishmaniasis linked to mixed infection by *L*. *infantum* and *L*. *major* was reported for an Iranian HIV-positive patient who did not respond to a different therapeutic scheme [39]. Coinfection by two trypanosomatids, *L. donovani* and *Leptomonas seymouri*, was detected in immunocompromised PDKL patients [99]. A coinfection by *L. infantum* and a *Crithidia*-related parasite may also be associated with fatal visceral leishmaniasis [100,101].

Mixed *Leishmania* spp. infections have also been demonstrated in domestic hosts and reservoirs. Mixed infection by *L. infantum* and *L. braziliensis* has been reported in horses, dogs and synanthropic rodents. Nevertheless, whether such infections impact the epidemiology of leishmaniasis in urban areas has not been clarified [25,32,36,38]. Mixed infection with *L. amazonensis* and *L. braziliensis* in dogs has also been observed in an urban area endemic for visceral leishmaniasis [42] and mixed infection caused by other *Leishmania* species and a variety of *Trypanosoma* spp. [41,102]. Furthermore, naturally infected dogs are prone to multiple *L. infantum* genotype infections [45]. However, whether these infections could impact the course of the disease or other characteristics, such as parasite transmissibility, remains to be elucidated.

## 3. Coculture and Experimental Mixed Infections by *Leishmania* Species and Their Interactions

Microorganisms live in communities and thus present broad inter- and intraspecies interactions. These interactions can be beneficial or harmful and can influence the fitness of such microorganisms. Whether these interactions are neutral, competitive or cooperative will depend on several factors, including the genetic background of the interacting microorganisms. Cooperative behavior provides a direct or indirect benefit to organisms [103,104] and is likely to occur among closely related microorganisms. Competition is more expected among distantly related microorganisms [105] and may impact virulence [106]. For bacterial species, physical or chemical contact often changes the phenotype, thus allowing for competition, mutualism or commensalism, and these relationships may have influenced their evolution [107,108].

Several studies have addressed the interactions among coinfecting parasite species, strains, or genotypes; however, only a few have investigated intra- and interspecies interactions. Most previous studies have focused on describing the interactions, while few have reported on the underlying mechanisms and consequences. Interactions among coinfecting parasitic species represent a relevant mechanism to maintaining genetic variation [109].

In the late 1980s, a report suggested that factors excreted by *L. amazonensis* could inhibit the in vitro growth of *L. mexicana* promastigotas [10]. These species present similar growth patterns in the absence of metabolic competition. Differential abilities to overcome environmental conditions were observed in cocultures of *L. donovani* and *Leptomonas seymouri* obtained after parasite isolation from PDKL patients, and variations in the culture media conditions enabled *L. seymouri* elimination [110]. In a multiwell plate system, *L. amazonensis* strains resistant to pentamidine inhibited the in vitro growth of nonresistant lineages [11], suggesting that secreted factors present in the shared culture medium rather than physical contact led to such alterations.

Mixed cultures of *L. donovani* strains with different drug resistance levels demonstrated increased fitness in drug-resistant parasites compared to more susceptible parasites, and they also presented higher tolerance to stress conditions [111]. In *Leishmania* spp., many drug resistance mechanisms are concomitantly associated with higher virulence or superior redox resistance, which may result in independent phenotype selection not associated with drug pressure, thus leading to the emergence of resistant strains among parasitic populations [112].

Competition among *L. major* clones derived from the same strain has been demonstrated. Initially, a more virulent clone represented the dominant competitor in the mixed culture; however, after one month of culture, the more attenuated clone was the predominant clone. Culture and environmental conditions, such as pH, change over time and could lead to the superior growth of clones with greater tolerance to these conditions. Nutrient requirements could explain the differences between the two clones, and the more virulent clone could buffer the media, thereby creating appropriate conditions for adaptations in the more attenuated clone [113]. Mixed multiclonal infections by *L. infantum* show that the phenotype of the virulent clone was dominant relative to the phenotypes of the associated low-virulence clones. After a challenge with homologous or heterologous strains or clones, virulent phenotypes were conserved and expressed in naive mice independent of the preexisting parasite population.

Studies on experimental mixed infection by *Leishmania* species are scarce. The few studies on this topic point to an important impact when two *Leishmania* species are coinfecting vertebrate hosts. Experimental infection of gerbil (*Rhombomys opimus*) with *L*. *major* and *L*. *turanica* led to a persistent infection that can reach up to 18 months, while separate infection with these species remained in the skin of the gerbil, which is a natural host, for up to six months at most. Such synergy thus favors the maintenance of *L. major* from the transmission season until the next [1]. Experimental infections in the sandfly *Phlebotomus papatasi* showed that *L. turanica* and *L. major* are able to develop together and do not show signs of competition [2]. Thus, the ability of *L. major* and *L. turanica* to participate in mutualistic interactions in the insect vector would have an impact on the transmission of these parasites to the vertebrate host. Together, these characteristics have a relevant impact on the epidemiology of cutaneous leishmaniasis caused by *L. major*.

Concomitant experimental infections with *Endotrypanum* and *L. guyanensis* showed different patterns compared to single infections, and although the authors suggested that the presence of *L. guyanensis* would inhibit the development of *Endotrypanum*, all cultivated samples of the parasites recovered from infected flies were characterized as *Endotrypanum*, which is a parasite that grows faster than *L. guyanensis* in vitro [114]. Experimental coinfections and single infections by *L. (L.) infantum* and *L. (V.) braziliensis* were performed in *Lutzomyia longipalpis* and *Lutzomyia migonei* [44]. Infections by *L. (L.) infantum* reached higher rates and grew more vigorously than that of *L. (V.) braziliensis*. Typical suprapylarian and peripylarian development were observed for *L. infantum* and *L. braziliensis*, respectively, as expected. Both *Leishmania* species completed their life cycle and produced infective forms in both sand fly species studied. The same results were obtained in coinfection experiments, demonstrating that the two parasites conclude their development and do not compete.

A comparison of a single infection of macrophages (lineage U-937) showed that the infectivity of *L. amazonensis* was higher than that of *L. infantum*, and this result was maintained with the dominance of *L. amazonensis* in the coinfected macrophages; however, a small portion of macrophages presented more than four *Leishmania*, which were rarely from different species [3]. In golden hamsters, mixed infection with *L. amazonensis* and *L. infantum* was associated with more severe disease than single infections. This result suggests that mixed infections could better suppress host immunity, thus allowing the parasites to multiply and impair macrophage effector function [4]. This study showed an earlier increase in the spleen in mixed infections, which was probably as a result of *L. amazonensis* dissemination, although in later stages of infection, the authors detected *L. infantum* outcompeting *L. amazonensis*.

The difference in fitness among lineages of *L. donovani* with diverse drug resistance patterns was demonstrated using experimentally mixed cultures. Competition was not observed when experimentally resistant promastigotes of *L. donovani* were cocultivated with susceptible lines. However, resistant lineages were more tolerant when mixed cultures were subjected to diverse stress conditions [111]. These results indicate that resistant phenotypes in *Leishmania* may be associated with the greatest in vitro fitness rather than a fitness cost, as observed in other models [115].

Mixed infections in vertebrate hosts may occur within the same tissue or even in the same cell. In the case of *Leishmania* spp., a parasite in the parasitophorous vacuoles (PVs) of macrophages, there is also the possibility of mixed infection in the same vacuole [46]. It is known that *L. amazonensis* is able to enter *Coxiella burnetti* vacuoles and then survive, differentiate, and replicate therein [116,117,118]. Furthermore, it has been shown that the large adaptive vacuoles of *L. amazonensis* are permissive to *T. cruzi* survival and differentiation and that noninfective epimastigotes are saved from destruction within the chimeric PVs [116,117,118]. The large vacuole that houses the *L. mexicana* species complex can explain why this multiparasite interaction is not observed under other conditions. For example, a mixed infection of macrophages by *L*. *infantum* and *Toxoplasma gondii* showed that they share the same macrophage but not the same PV [116,117,118]. A study of mixed infection in macrophages by *L. amazonensis* and *L. major* found no fusion of PVs containing both amastigotes. Interestingly, PVs containing *L. major* promastigotes fused with preestablished *L. amazonensis* PVs. In these chimeric vacuoles, *L. major* promastigotes remained motile and multiplied but did not differentiate into amastigotes [28]. Considering the *Leishmania*–macrophage interaction, species-specific differences were demonstrated in the biogenesis of the PV. For example, amastigotes from the *L. mexicana* species complex use large vacuoles, which may contain many parasites, while a single *L. major* amastigote occupies a smaller tight PV [119]. The presence of both *L. amazonensis* and *L. mexicana* within the same communal vacuole has also been described [46].

## 4. Do Coinfections Promote Hybrid Formation?

Genotype, strain, and species interactions among *Leishmania* parasites can occur in both vertebrate and invertebrate hosts. Nevertheless, genetic exchange has been mainly demonstrated to occur in sand fly vectors, with experimental evidence of intraspecific hybrids of *L. major*, *L. tropica*, and *L. donovani* [29,31,43], cross-species hybrids between *L. major* and *L. infantum* [37], and intraclonal or selfing hybrids of *L. infantum* [35]. Although few studies have investigated hybrid formation during vertebrate infection [27], a study that performed DNA quantification showed that infected macrophages could harbor 4N amastigotes, suggesting that genetic exchange is possible in mammalian host cells [120]. Recently, the possibility of intraclonal and interspecific genetic exchange among parasites of the *L. mexicana* complex was explored, and unlike other *Leishmania* species, the species of this complex replicate in spacious communal vacuoles that may provide an environment favorable to genetic exchange, although the resulting products of those putative genetic events were unstable [46].

Cell fusion in *L. infantum* and *L. tropica* was observed following promastigote axenic in vitro culture. Fusion began with an attachment of the posterior extremities of two ovoid flagellates, which was followed by complete fusion, with the disappearance of adjacent cell membranes and the appearance of a larger and shorter flagella [121]. Evidence of sexual reproduction in *Leishmania* was also quantitatively demonstrated through microspectrophotometric analyses of nuclear fusion in the intracellular form, i.e., the amastigote, within the mammalian host [120]. Furthermore, fluorescence microscopy showed that *L. donovani* hybrids occurred in experimentally infected sandflies, although the hybrids were not viable in vitro [29].

Broad agreement has been reached that *Leishmania* possesses the machinery for genetic exchange, and the debate regarding reproductive strategies pertains mainly to the frequency of sexual recombination and its impact on population structure. To date, the most accepted environment for generating *Leishmania* hybrids is inside the invertebrate host among promastigotes. Double-drug-resistant clones could be generated by coinfecting sand flies with various pairwise combinations of parental lines expressing distinct drug-resistant markers. In almost every case, these clones appeared to be full genomic hybrids based on their biparental inheritance of allelic markers distributed across the nuclear genome [27,31,43]. The parental chromosome contributions were consistent with a meiotic process, and deep sequencing of backcross progeny clones revealed genome-wide recombination patterns, indicating that classic crossovers occur at meiosis [43].

Several studies have isolated strains characterized as putative hybrids between different *Leishmania* species. They are most common hybrids between closely related species, such as some dispersed on the American continent, namely, *L. braziliensis* and *L. panamensis* [122], *L. braziliensis* and *L. peruviana* [123], *L. braziliensis* and *L. guyanensis* [124], *L. naiffi* and *L. lainsoni* [125], and recently natural *L. guyanensis/L. shawi* hybrids were isolated from patients with American Cutaneous Leishmaniasis in the Amazon region of Brazil [126]. Hybrids between closely related species from the Old World were also described, namely, *L*. *major* and *L. arabica* [127,128]. Putative hybrids between *L. major* and *L. infantum*, which are phylogenetically distant species with different vectors and reservoir hosts, have also been described [129] but less frequently. The fitness of *L. major*/*L. infantum* hybrids was increased when compared with that of *L. infantum*. Genetic exchange appears to have conferred a certain level of *L. major* lipophosphoglycan (LPG) to the mentioned hybrids, thus enabling them to survive in the specific vector *P. papatasi*, which is permissive to *L. major* but not to *L. infantum* [130]. In addition to altered transmission capabilities or the production of a more aggressive infection [131], the consequences of genetic exchange may have many kinds of epidemiological significance. Of note is the outbreak of CL in Peru in the 1990s, which was associated with the F1 hybrids of *L. braziliensis* and *L. peruviana* [132]. Indeed, genetic exchange might facilitate the emergence and spread of new phenotypic traits [133].

Beyond the observations of hybrids between *Leishmania* species and strains, recent studies have reported mito-nuclear discordance among *Leishmania* strains, but it is not clear how this occurs and if it is the same mechanism of formation of nuclear DNA hybrids [134]. Analysis of complete genome sequences of a large sample of *L. braziliensis* and *L. peruviana* strains from Peru showed evidence of meiotic-like recombination between *Leishmania* species, resulting in full-genome hybrids. Analysis of the mitochondrial genome of hybrid strains indicated that they consisted of homogeneous uniparental maxicircles but minicircles derived from both parental species [132].

## 5. The Occurrence of a Subpopulation of Parasites within One Strain—Aneuploid Mosaicism and Haplotype Selection/Fluctuation: Already a Mixed Content?

Various studies have reported in recent years that one *Leishmania* isolate is composed of cells presenting different homologous chromosome contents and variable gene copy numbers. This feature was better characterized by FISH in 2011 and is referred to as aneuploidy mosaicism [135]. Further studies demonstrated that the aneuploidy profile of an isolate might change as a consequence of environmental conditions, which is a reflection of this strategic adaptations harbored by *Leishmania* parasites [136]. Such plasticity of genes and whole chromosome copy numbers directly affects the parasite transcriptome. Thus, same-strain phenotypic variances are likely to occur depending on the fluctuation of these subpopulations of cells carrying distinct genome contents.

Based on the above statements, we assume that aneuploidy mosaicism introduces complexity to discussions of multiple infections in *Leishmania*. This mosaic feature implies that each strain is already a mixture in a sense, which represents a strategy of the parasite to balance short-term and long-term adaptation [137]. Selection of haplotypes that results in allelic frequency modification (haplotype fluctuation:) and karyotype fluctuation (implies a preexistence of karyotypic mosaicism of the population of parasites in a given condition, e.g., population of parasites maintained in culture, parasites in the infection of their hosts) that may be of benefit to the parasite will ultimately maintain the variability and potentially promote phenotypic variance in the *Leishmania* isolate. Therefore, a given isolate might find different solutions for environmental challenges, such as drug exposition and in vitro culture. Considering the present discussion in this review, we believe that new single-cell-based techniques will be able to reveal the effects of a subpopulation of cells based on tracking and determining the genome content individually [138]; moreover, such techniques will contribute to mapping the interaction between parasite cells, either from different species/strains or within a given isolate.

Another critical point regarding plasticity is the genetic fluctuation in the leishmanial strain. This parasite is unique in its ability to increase its genetic diversity, in which both the karyotype and the number of haplotypes are changed. *L. donovani* promastigote strains isolated from golden hamsters were compared among early and late passages. Fluctuations in the allele frequency were observed along the passages, indicating mosaic aneuploidy in different combinations. Only 10% of the 204 observed karyotypes showed a high frequency. In vivo analyses demonstrated different localizations of the aneuploid profile subpopulations in the liver and spleen. These results suggest that different alleles could be related to specific localizations in the host and represent the fitness of diverse subpopulations. Altogether, these observations may indicate that *Leishmania* spp. are able to change their genetic repertoire, thereby magnifying their ability to adapt to stressful and divergent environmental conditions, improving their survival, and increasing the diversity within the populations [137].

## 6. Intercellular Communication

*Leishmania* spp. are heteroxenous unicellular parasites. The survival achieved thus far by different species of this parasite is based on their successful morphological–biochemical–physiological adaptations, environmental sensing ability, molecular and genetic organization to optimize responses and interactions, and communication within contiguous populations (intra/interstrain and intra/interspecies). Many functions and molecules have been studied and are related to environmental sensing and adaptative responses, such as cAMP (cyclic adenosine monophosphate), inositol phosphatases, kinases, phosphoproteins and heat shock proteins in kinetoplastids. Related genes are usually involved in the influence of signal transduction on infectivity, cell growth, and differentiation. Approximately 6% of the genes localized on chromosomes 1 and 3 are associated with these processes [139].

In response to environmental challenges, single and multicellular organisms exhibit a conserved signaling pathway composed of surface receptors that transduce signals to kinases and phosphatases. Downstream, these cascades result in variations in gene expression and protein abundance, thus providing phenotypic variations [140]. In parasitic protozoa, the relationship between signaling pathway components and regulators is not fully understood [141]. Parasites have developed a range of mechanisms for communicating with each other, which sometimes occurs directly from parasite to parasite or is driven by the infected host cell—or components derived from it—as an intermediary. By emitting signals that can be dispersed within the host, parasites can also have wide-ranging effects on the course of an infection and its pathology. Intercellular communication mechanisms may rely on direct cell–cell contact or extracellular vesicles (EVs) for the transfer of secreted molecules [142]. Exosomes represent the smallest type of EV and may contain lipids, proteins, mRNAs and microRNAs [143]. Exosomes from *Leishmania* spp. contain chaperones (e.g., Hsp70), biogenesis (e.g., clathrin) and cytoskeletal proteins (e.g., actin, tubulin), toxins, virulence factors (e.g., GP63) and RNAs [144]. Secreted exosomes can be incorporated during parasite cell interactions, further inducing differentiation, changes in infectivity, etc. Therefore, EVs constitute a system of signal transference among cells [145].

In *T. brucei*, exosomes influence social motility by inhibiting parasitic growth under stressful conditions, thus leading to stress signal secretion for contiguous parasites [146]. It was demonstrated that purified exosomes derived from drug-resistant *L. infantum* strains (resistant to antimony, miltefosine or amphotericin B) differed in the content composition, size, distribution and morphology. These mechanisms might be shared within the parasitic population, possibly resulting in increased survival and resistance to other stressful conditions [147]. Moreover, the endosymbiont *Leishmania* RNA virus (LRV), which has been related to worsening disease prognosis [148], exploits the exosome pathway to transmit the viral particle from one parasite to another [15].

There are mechanisms that enable a population of individual cells to coordinate global behavior and act as a multicellular unit, which is a phenomenon known as quorum sensing (QS). Microorganisms may coexist in narrow associations, where they interact and communicate with each other to better adapt to the environment and coordinate each other’s functions within their respective niches. Intercellular communication may occur by genetic or biochemical transfer that may be mediated by vesicles [149]. QS in trypanosomatids has been studied among inter- and intraspecies. *T. congolense* was able to promote differentiation to the stumpy forms of *T. brucei* in vitro, while in vivo coinfection accelerated the stumpy form of *T. brucei* differentiation, resulting in lower parasitemia. This effect was lost when the QS pathway was compromised by the silencing of TbHYP2. TbHYP2 was previously identified as part of the *T. brucei* QS pathway [17,150].

QS in *T*. *brucei* is associated with different factors, such as small secreted molecules, stump induction factor (SIF), flagellar motility and some specific genes. SIF and flagellar signaling are associated with the cAMP cascade in different forms directly associated with social motility, thus influencing parasitemia. cAMP is produced by receptors of adenylate cyclase (AC), and at the parasitemia peak, the levels of cAMP increase approximately three times. In contrast, cAMP decreases significantly during the transition to the stumpy form [150,151,152]. Flagellar phosphodiesterase PDEB1 is related to restricted and local cAMP production by AC [153]. Recently, it was demonstrated that PDEB1 is necessary for in vitro signals for social motility. Parasites lacking PDEB1 displayed increased levels of cAMP in the flagellum and cell, and they could not produce localized cAMP and respond to the signals associated with peritrophic matrix crossing, which would result in impaired vector colonization.

Another signaling mechanism was associated with the atypical kinase DYRK, which has been identified and described as an important component of the QS cascade in *T. brucei* and perhaps in trypanosomatids since *Leishmania* spp. orthologs were also identified [154]. The DYRK family in *Leishmania* spp. consists of eight members. DYRK1 was implicated in stationary-phase survival and infectivity and localized in the flagellar pocket area (strongly associated with QS in trypanosomatids). Knockout of DYRK1 in *L. infantum* led to an increased proliferation rate in the logarithmic phase compared to the wild type, and the overexpression of this gene resulted in decreased proliferation. During the stationary phase, knockout was morphologically and biochemically distinct from that of the wild type, exhibiting a rounded shape and a cytoplasm with intense vacuolization, lipid body accumulation and a switch in the ratio of saturated/polyunsaturated lipids. Finally, DYRK1 knockout influenced metacyclogenesis and dramatically reduced the performance in in vitro infection [155].

Noncoding RNA is a group of ribonucleic acid molecules comprising small nuclear RNA, small interfering RNA, long noncoding RNA and microRNA [156]. MicroRNA may be transferred by exosomes influencing host cells [157]. Bacterial noncoding RNAs are classified as small RNAs, and their role in QS has recently been suggested, especially for bacterial survival in harsh environments [158]. Noncoding RNA are among a small group of *Trypanosoma brucei* genes showing transiently increased transcript levels across the slender to stumpy transition point [159], but so far, researchers have not demonstrated the role of noncoding RNA in QS in any trypanosomatid. 

The majority of microRNA studies in *Leishmania* spp. are focused on host interactions and the immune response. Many studies have demonstrated that infection by *Leishmania* spp. influences the microRNA profile in the host (including macrophages and dendritic cells from humans, dogs and mice) in association with virulence factors [160]. The inhibition of some microRNAs reduced *L. braziliensis* growth earlier after in vitro macrophage infection [161]. The role of microRNAs in cross-*Leishmania* species (or strains or genotypes) communication is an open avenue to be explored, which might contribute to a better understanding of many biological processes occurring in the dynamic interaction among *Leishmania* parasites during vertebrate and invertebrate infections.

Intercellular communication includes the interplay of features according to a very intricate orchestra. Many factors may interact, resulting in a beneficial or negative relationship.

## 7. Conclusions

The change in *Leishmania* spp. fitness and behavior might be a result of the interplay among diverse factors. The parasitic genetic background is a source of phenotypic variability, which might be selected as an environmental change and challenge response. Host immunity may represent an important source of these challenges. The possibility of interactions within inter/intraspecies, genetic variability and intercellular communication might provide sources for enrichment in parasitic plasticity. In addition, amplified genetic polymorphisms, hybrid generation and phenotypic adaptations (e.g., behavior, fitness) may arise. Moreover, the complex balance of these multiple factors and features could lead to diverse disease outcomes. Multiple infections in either invertebrate or vertebrate hosts may correspond to diverse prognoses. Finally, deeper studies and a better understanding of these interactions are mandatory. In association with molecular tools, they may afford valuable methods of improving disease prognosis and drive better treatment designs.

## Figures and Tables

**Table 1:** Summary of studies reporting natural or experimental interactions among *Leishmania* species or strains.

Reference	Species and/or Strains Observed or Employed in the Study	Methodological Approach: Natural Infection; Experimental ^1^ Infection or Coculture	Host	Main Finding
Silveira et al., 1984 [18]	*L. braziliensis* and *L. amazonensis*	Natural	Human	First human case of mixed infection determined by *L. braziliensis* and *L. amazonensis*
Oliveira Neto et al., 1986 [19]	*L. braziliensis* and *L.* *donovani*	Natural	Human	Previous infection with one *Leishmania* species did not protect against infection with the other
Pacheco et al., 1987 [10]	*L. mexicana* and *L. mexicana amazonensis* ^2^	Coculture	-	Inhibition of one *Leishmania* species by exometabolites of another species
Mebrahtu et al., 1991 [20]	*L. donovani* and *L. major*	Natural and experimental	Human/Syrian hamsters and BALB/c mice	Mixed cultures obtained from spleen fragments were inoculated in laboratory animals and produced both visceral and cutaneous leishmaniasis
Al-Diwany et al., 1995 [21]	*L. donovani* and *L. major*	Natural	Human	Observation of concomitant visceral and cutaneous leishmaniasis
Abdullah et al., 1998 [3]	*L. mexicana amazonensis*^3^*, L. donovani* and *L. infantum*	Experimental	Human monocyte cell line—U-937	Preinfecting with one *Leishmania* species did not exclude the infection by a second species added
Agnew et al., 2001 [11]	*L. amazonensis* strains: LaR5CL1 and LaR20CL1	Coculture	-	Interactions among strains influenced in vitro growth of *Leishmania amazonensis*
Strelkova et al., 2001 [1]	*L. major* and *L. turanica*	Natural and experimental	Gerbil—*Rhombomys opimus*	Alteration in the transmission dynamics
Martinez et al., 2002 [22]	*L. amazonensis* and *L. infantum*	Natural	Human	Cutaneous lesion presenting atypical characteristics, possibly due to coinfection
Bastrenta et al., 2003 [23]	*L. braziliensis* and *L. mexicana / Leishmania* spp. and *T. cruzi*	Natural	Human	Unexpected therapeutic outcomes which were speculated to be associated to the mixed infection
Antoniou et al., 2004 [6]	*L. infantum* strains: zymodeme MON-98 and zymodeme MON-1	Natural	Human	Unexpected therapeutic outcomes which were speculated to be associated to the mixed infection
Porrozzi et al., 2004 [24]	*L. amazonensis, L. guyanensis*, *L. major* and *L*. *braziliensis*	Experimental	Rhesus monkeys—*Macaca mulatta*	Cross-reacting immune responses and possible cross-protection between taxonomically different *Leishmania* species
Madeira et al., 2006 [25]	*L. braziliensis* and *L. chagasi* ^4^	Natural	Canids—*Cannis familiaris*	First case of coinfection with *L. braziliensis* and *L. chagasi* ^4^ in a naturally infected dog from Rio de Janeiro, Brazil
Mahmoudzadeh-Niknam et al., 2007 [26]	*L. tropica* and *L. major*	Experimental	BALB/c mice	Primary infection with *L. tropica* induces partial protection against *L. major* infection
Akopyants et al., 2009 [27]	*L. major* strains	Experimental	Sand flies—*P. duboscqi*	Evidence that *Leishmania* promastigotes are capable of a sexual cycle consistent with a meiotic process
Real et al., 2010 [28]	* L. amazonensis * and *L. major*	Experimental	BALB/c mice’s bone marrow-derived macrophages	Parasitophorus vacuoles (PVs) presumably customized by *L. major* amastigotes or promastigotes, differ in their ability to fuse with *L. amazonensis* PVs
Sadlova et al., 2011 [29]	*L. donovani* strains carrying hygromycin or neomycin resistance genes	Experimental	Sand flies—*P. perniciosus* and *L. longipalpis*	Experimental evidence of intraspecific hybrids
Santos-Oliveira et al., 2011 [30]	* L. infantum * strains: zymodeme MON-1, type A (bone marrow) and *L. donovani* zymodeme MON-2 (skin)	Natural	Human	Atypical cutaneous lesions appearing after long-term evidence of visceral parasites
Chajbullinova et al., 2012 [2]	*L. major* and *L. turanica*	Experimental	Sand flies—*P. papatasi*	*L. turanica* and *L. major* are able to develop in *P. papatasi* together, without any visible sign of competition; no hybrids detected
Shirian et al., 2012 [5]	*L. tropica* and *L. major*	Natural	Human	Previous infection with one *Leishmania* species did not protect against infection with the other
Inbar et al., 2013 [31]	*L. major* strains: obtained from differents hosts	Experimental	Sand flies—*P. duboscqi* and *L. longipalpis*	Experimental evidence of intraspecific hybrids
Soares et al., 2013 [32]	*L. infantum* and *L. braziliensis*	Natural	Equine—*Equus caballus*	First mixed infection of *L. infantum/L. braziliensis* in equine reported in the world
Veland et al., 2013 [33]	*L. braziliensis* and *L. laisoni*	Natural	Human	Good response to treatment and no evidence of mucosal involvement
Babiker et al., 2014 [34]	*L. donovani* and *L. major*	Natural	Human	No evidence of visceralization observed, despite the infection by *L. donovani*
Calvo-Álvarez et al., 2014 [35]	*L. infantum*: transgenic lines expressing drug resistance markers	Experimental	Sand flies— *P. perniciosus*	First evidence of intraclonal genetic exchange between two *L. infantum lines*
Pires et al., 2014 [36]	*L. braziliensis* and *L. chagasi* ^4^	Natural	Canids—*Cannis familiaris*	Reinforces the importance of using serological and molecular techniques in the epidemiological surveillance of canine populations in endemic areas
Romano et al., 2014 [37]	*L. major* and *L. infantum*	Experimental	Sand flies—*L. longipalpis*	First experimental confirmation of cross-species mating in *Leishmania*
Ferreira et al., 2015 [38]	*L. infantum* and *L. braziliensis*	Natural	Rodents—*Mus musculus* and *Rattus rattus*	First description of mixed infection by *L. braziliensis and L. infantum* in rodents caught in an urban area
De Lima Celeste et al., 2017 [4]	*L. amazonensis* and *L. infantum*	Experimental	Syrian hamster—*Mesocricetus auratus*	Mixed infections were associated with more severe clinical manifestations than single infections
Badirzadeh et al., 2018 [39]	*L. infantum* and *L. major*	Natural	Human	Influence in the therapeutic response
Gosch et al., 2018 [40]	*L. guyanensis* and *L. amazonensis*	Natural	Human	Mixed clinical, histopathological and immunological characteristics related to the two species
Herrera et al., 2018 [41]	*L. mexicana mexicana*^5^*/L. braziliensis braziliensis*^6^; *L. mexicana mexicana*^5^*/T. cruzi*, and *L. braziliensis braziliensis*^6^*/T. cruzi*	Natural	Canids—*Cannis familiaris*	Coinfection by different *Leishmania* species and by *T. cruzi* and *Leishmania* spp. in dogs from Mexico
Alves Souza et al., 2019 [42]	*L. amazonensis, L. braziliensis* and *L. infantum*	Natural	Canids—*Cannis familiaris*	Coinfection by different *Leishmania* species in dogs from Minas Gerais, Brazil
Inbar et al., 2019 [43]	*L. major* strains: obtained from differents hosts/ *L. tropica* strains: obtained from differents hosts	Experimental	Sand flies—*P. duboscqi* and *L. longipalpis*	Experimental evidence of intraspecific hybrids
Alexandre et al., 2020 [44]	*L. braziliensis* and *L. infantum*	Experimental	Sand flies—*L. migonei* and *L. longipalpis*	Mixed infection did not affect each parasite development and no competition was observed
Cupolillo et al., 2020 [45]	*L. infantum*: multiple genotype	Natural	Canids—*Cannis familiaris*	Multiple genotype infections occur within a single host and tissue
Telittchenko and Descoteaux 2020 [46]	*L. mexicana* and *L. amazonensis*	Experimental	C57BL/6 mice and bone marrow-derived macrophages from C57BL/6 mice	Evidence of not sustained genetic exchange in both axenic promastigote cultures and infected macrophages

^1^ in vivo assays using vertebrate or invertebrate hosts and/or cell lines; considering the actual classification for *Leishmania* species: ^2^ *L. mexicana* and *L. amazonensis*; ^3^ *L. amazonensis*; ^4^ *L. infantum*; ^5^ *L. mexicana;* ^6^ *L. braziliensis.*

**Table 2:** Classification of *Leishmania* species (Class Kinetoplastea; Order Trypanosomatida; Family Trypanosomatidae; Subfamily Leishmaniinae), excluding synonyms, *nomen nudum* and those not completely classified.

Genus	Subgenus	Species ^1^	
*Leishmania*			
	*Leishmania*		
		L. donovani * Laveran & Mesnil 1903 [50]	L. tropica * Wright 1903 [51]
		L. infantum * Nicolle 1908 [52]	L. major * Yakimoff & Schokhor 1914 [53]
		L. archibaldi * Castellani & Chalmers 1919 [54]	L. mexicana * Biagi 1953 [55]
		L. gerbilli * Wang et al., 1964 [56]	L. amazonensis * Lainson & Shaw 1972 [57]
		L. aethiopica * Bray et al., 1973 [58]	*L. aristidesi* Lainson & Shaw 1979 [59]
		L. venezuelensis * Bonfante-Garrido 1980 [60]	L. killicki * Rioux et al., 1986 [61]
		L. arabica * Peter et al., 1986 [62]	*L. turanica* Strelkova et al., 1990 [63]
		*L. forattinii* (oshida et al., 1993 [64]	L. waltoni * Shaw et al., 2015 [65]
	*Viannia*		
		L. braziliensis * Vianna 1911 [66]	L. peruviana * Velez 1913 [67]
		L. guyanensis * Floch 1954 [68]	L. panamensis * Lainson & Shaw 1972 [57]
		L. lainsoni * Silveira et al., 1987 [69]	L. shawi * Lainson 1989 [70]
		L. naiffi * Lainson & Shaw 1989 [71]	L. lindenbergi * Silveira et al., 2002 [72]
		*L. utingensis* Braga et al., 2003 [73]	
	*Sauroleishmania*		
		*L. tarentolae* Wenyon 1921 [74]	*L. hemidactyli* Mackie et al., 1923 [75]
		*L. ceramodactyli* Adler & Theodor 1929 [76]	*L. nicollei* Khodukin & Sofiev 1940 [77]
		*L. gymnodactyli* Khodukin & Sofiev 1947 (apud Killick-Kendrick 1986 [78])	*L. adleri* Heisch 1958 [79]
		*L. hoogstraali* McMillan 1965 [80]	*L. senegalensis* Ranque 1973 [81]
		*L. gulikae* Ovezmukhammedov & Saf’janova 1987 [82]	
	*Mundinia*		
		*L. enriettii* Muniz & Medina 1948 [83]	L. martininquensis * Debois et al., 2014 [84]
		*L. macropodum* Barratt et al., 2017 [85]	L. orientalis * Jariyapan et 2018 [86]
	*Porcisia*		
		*L. hertigi* Herrer 1971 [87]	*L. deanei* Lainson & Shaw 1977 [88]

^1^*L. herreri*, *L. colombiensis* and *L. equatorensis* are not reported in this table since for some authors, they are classified as *Endotrypanum*; * *Leishmania* species detected causing human diseases.
